# Morgan County Medical Society

**Published:** 1868-03

**Authors:** C. T. Wilbur

**Affiliations:** Secretary


### MORGAN COUNTY MEDICAL SOCIETY.

The Morgan County Medical Society met on Thursday, December 12th, at 2½ o'clock P.M., at the Infirmary on South Sandy Street, with Dr. Henry Jones, the President, in the Chair. The proceedings of the last meeting were read and approved.

A motion was carried "that the names of applicants for admission to the Society be handed by the Secretary to the Committee on Membership."

The regular exercise for the day was then taken up. A verbal report upon the subject of Typhoid Fever, by Dr. Henry Jones. He could not be expected to say very much upon the subject in the short time allotted to him, but would endeavor to touch upon a few of the important points connected with the treatment of this disease.

The dearth of knowledge on the pathological conditions in fevers afforded us by the writers of the early part of this century, was commented upon, when the doctor gave his views upon certain points in the treatment of typhoid fever:

Little internal but active external treatment was the order for many years in France. Some recommended bleeding—others purging, and that only. I would first suggest, in relation to my own treatment, that particular attention be paid to the thorough ventilation and cleanliness of the sick room, and to the procuring of a good nurse. There should be no carpet in the room, and no draught of air over the bed of the patient. Activity of practice is not needed. Typhoid fever is often treated without being recognized. The peculiar rose-colored spots are generally present. There is always tenderness in the right iliac region. There may be no diarrhœa. I am prejudiced in favor of the old treatment, also of emetics, though they are now out of date. I think it would be well to use many things now out of date. In regard to purgatives, I would say that I do not think they interfere with the abnormal condition of Peyer's glands injuriously, but that they are relieved by keeping the intestines in a clean state. I employ mercurial purgatives in the first days of the disease, and in some instances occasionally in the farther progress of the disease.

Bleeding may sometimes be of benefit in typhoid fever. I have never been sorry when I have bled in this disease, but have sometimes been sorry that I have not bled. The famous French physicians were in favor of bleeding in the first stage. I do not think many patients are hurt by it in the first stage. I predict that hereafter more bleeding will be done than in the last ten years. I think the profession is veering round. There has been no change in the type of typhoid fever, but there has been a complete change of practice. I do not fear the diarrhœa of typhoid fever; fæcal matter I consider a source of irritation; with gentle purgative remedies, I believe you will have no diarrhœa. I do not believe in the use of astringents. Do not be too sedulous to diminish the diarrhœa in this disease. We sometimes have hemorrhage. I place much reliance upon ice-water injections; I also use gallic acid and sugar of lead. I often use pediluvia, sinapisms, opium, and tartar emetic. I use stimulants a great deal less than I did ten years ago. Stimulants have been very fashionable of late; stimulation in everything has been the order of the day. I think the profession are altering their views upon this point. I rarely give stimulants in the early stages of this disease. Where the patients like stimulants, and they are craved, I should use them, but not otherwise. Many of the best methods of practice have become obsolete, and many of the old customs of our fathers are occasionally revived as new practice. I have not time to say anything of diet. I have seen but three cases of perforation of the intestines; they all died. To recapitulate, briefly: I suggest, as treatment, bleeding, purgatives, and do not be over alarmed about diarrhœa—do not lock up the system.

Dr. Long had observed very excellent results from the ice-water injections in hemorrhage, and had been induced to believe that their application would be beneficial to the ulcerated condition of the intestines themselves.

Dr. Prince remarked: I have used the tinct. chloride of iron, and the sulphate of iron and capsicum, in this disease, with beneficial results.

Dr. Fisher remarked, that at the meeting of the State Medical Society, Dr. Noble, of Bloomington, stated that "typhoid fever was a disease of the bowels." What is it? Will the gentlemen give us their opinions as to the pathology of the disease?

Dr. Prince remarked, that, during the years 1861 and 1862, he had many cases of typhoid fever in military hospitals. We found ulcerations of the small intestines in all fatal cases. But the severity of the symptoms had but little to do with the extent, the number, and size of the ulcerations found upon *post mortems*. The eruption was generally observed, but not in all cases. In one case, I observed a condition of contraction of the intestine, caused by the cicatrices of the ulcerated patches of Peyer's glands, taken from a convalescent patient who died from eating an orange. The intestine was closed by stricture produced by these cicatrices. In my opinion, the ulcers result from the poison of the fever, and are not the cause of the fever. The relations between the ulcerations and the fever I regard as certain, as the relation between the eruption and the poison in small-pox. In some cases, there was a slough hanging, ready to be picked off, while, in other cases, the ulcerations were healed over with a very perceptible cicatrix. We examined some 8 or 10 cases. One case of typhoid fever got well, was discharged from the hospital, returned to duty, but was very soon taken with pneumonia, and died. The *post mortem* examination revealed in the intestines some cicatrices and some ulcerations that were not healed, showing that men were not always well when they were considered so.

Dr. Edgar remarked, that specimens of pieces of the intestines of typhoid fever cases and of chronic diarrhœa cases, when compared, presented very much the same appearance. Referred to an epidemic of both these diseases, at the same time, at Vicksburgh, during the war. At the *post mortem* examinations in these cases, no sloughs were observed, but a ragged condition, as if sloughing had taken place, was observed in both diseases. We found well-marked cases of typhoid fever with no ulcerations of the bowels.

Dr. Prince remarked, that he had one fatal case of fever at the hospital before spoken of, in which there was no diarrhœa, but in this case he found more ulcerations than in any other case examined.

Dr. Henry Jones remarked, that where, in life, unequivocal symptoms of typhoid fever existed, after death, *post mortem* examinations always revealed local lesions of the intestine, or, at all events, so the profession abroad had decided.

Dr. Edgar stated, that he did not believe that there was a specific *materies morbi* necessary for the production of typhoid fever. He believed that a combination of causes affecting the nervous centres sometimes produced it. The Doctor read the statistics of a series of years of the results of the cases occurring at the Massachusetts Hospital, at Boston, showing different results during the different years, as he presumed, under the same treatment. His inference was, that epidemics of typhoid fever varied in intensity, and some were much more fatal than others, no matter what the mode of treatment adopted.

Dr. Prince suggested that Dr. Austin Flint recommended sulphuric acid as a remedy. Had more faith, however, in fresh air, in tents, in warm weather. Dr. Flint reduced the mortality by this treatment in the open air, to at least ten per cent.

Dr. Fisher thought we ought to understand the pathology of the disease, before we could get at a proper line of treatment; for a variety of opinions had been expressed as to its cause. Some considered that the disease arose from ulcerations of the intestines; others, that the fever produced the ulcerations; others, that a combination of causes produced the disease.

Dr. Henry Jones remarked, that a distinction must be made between typhoid symptoms and the specific disease typhoid fever.

Dr. Prince said that Dr. Woodward had advocated the existence of a typho-malarial fever, which yields more readily to quinine, and this remedy is usefully employed where it could not be in typhoid fever.

Dr. Edgar suggested that flatus was a triumph of chemical over vital causes. A bougie might be employed to relieve it, but, as suggested by Dr. Samuel Adams, care should be taken to place the patient upon the right side during the process. He had used pills of nitrate of silver as a remedy in ulceration.

Dr. H. K. Jones, of Jacksonville, and Dr. Kimber, of Waverly, were appointed for essays at the next meeting.

At 5 o'clock P.M., the Society adjourned, to meet on the second Thursday in January, 1868.

C. T. WILBUR, M.D., *Secretary.*

---

Society met at 2½ o'clock P.M., at the Court House in Jacksonville, on Monday, January 9th, 1868.

Dr. S. S. Nesbitt, of Virginia, Cass County, was elected a member of the Society.

Dr. Henry Jones reported a very rare case, which had recently occurred in his practice, and was requested by the Society to send the same to some medical journal for publication, as a contribution from this Society.

Dr. H. K. Jones, who was the appointee for the regular exercises of the day, read an interesting essay on the Function of the Spleen, and was also requested to contribute the same to some medical journal, as an emanation from this Society.

Dr. Edgar remarked that the essay was very interesting to him, by reason of its connection with the topic of the last month—for Dr. Bennett, of Edinburgh, had observed that the spleen was larger and more often diseased in typhoid fever than any other organ. Dr. Bennett's definition of typhoid fever is, "an essential disease, dependent upon some unknown constitution of the blood, the local lesions being consequential." In two fatal cases that were carefully observed by Dr. Bennett, no rose-colored spots were found. That seven out of sixty cases exhibited no lesions of structure, on *post mortem* examinations. The spleen was more constantly affected than any other organ of the body. He tried quinine, as a remedy, in eighteen or nineteen cases, and concluded that it could not be relied on.

Dr. Prince thought that typhoid fever might arise from air-poisoning, by the reconsumption of emanations from the body.

Dr. Henry Jones said that he did not think that the authorities and opinions offered by Drs. Edgar and Prince warranted us in discarding the old recognized ideas in relation to typhoid fever. I believe that we should occupy this ground: that it is an essential disease, produced by a particular agent; that the lesion of Peyer's glands are the almost invariable expression of the disease; and, also, that almost always appear the rose-colored spots.

Drs. Kellogg and King were appointed essayists for the next meeting.

At 5 o'clock, adjourned, to meet on the second Thursday of February. C. T. WILBUR, M.D., *Secretary.*

